# Never tested for HIV? Characteristics of first-time testers among MSM in Chongqing and Chengdu, China

**DOI:** 10.3389/fpubh.2026.1863281

**Published:** 2026-08-12

**Authors:** Liping Fei, Jing Lin, Chao Zhou, Wei Zhang, Xu Jingpei, Li Yan, Rui Zhang, Hua Zhao, Wei Huang, Rongrong Lu

**Affiliations:** 1 Chongqing Center for Disease Prevention and Control (Chongqing Academy of Preventive Medicine), Chongqing, China; 2 Chengdu Center for Disease Prevention and Control, Chengdu, China; 3 Chongqing Shapingba Center for Disease Prevention and Control, Chongqing, China; 4 Chongqing Nana Center for Disease Prevention and Control, Chongqing, China

**Keywords:** AIDS-acquired immunodeficiency syndrome, first-time testing, HIV, men who have sex with men (MSM), sexual health

## Abstract

**Background:**

Men who have sex with men (MSM) are disproportionately affected by HIV, and initial HIV testing is a critical entry point for early diagnosis and treatment. However, factors associated with first-time HIV testing among MSM in the Chengdu-Chongqing region remain unclear. This study aims to assess HIV testing coverage and identify factors associated with first-time HIV testing among MSM in this region, thereby providing scientific evidence to help achieve the UNAIDS 95-95-95 targets, which include diagnosing 95% of people living with HIV by 2030.

**Methods:**

This cross-sectional study was conducted through community-based organizations in Chengdu and Chongqing from August to December 2024. Data on socio-demographics, sexual behaviors, sexually transmitted infection (STI) history, new psychoactive substance (NPS) use, and HIV testing history were collected using a structured questionnaire. Chi-square tests and multivariable logistic regression were employed to identify factors associated with first-time HIV testing.

**Results:**

Of the 514 MSM included, 156 (30.4%) were identified as first-time testers. Multivariable logistic regression revealed that the absence of group sex in the past month was associated with an increased likelihood of first-time testing (aOR = 3.22, 95% CI: 1.08–9.56). In contrast, MSM with a college degree or higher (aOR = 0.46, 95% CI: 0.23–0.93) and a history of STI in the past year (aOR = 0.13, 95% CI: 0.02–0.99) were associated with a decreased likelihood of first-time testing.

**Conclusion:**

Approximately one-third of MSM in Chengdu and Chongqing have never been tested for HIV. We recommend enhanced, targeted interventions for lower-educated MSM, along with integrated HIV and STI testing services, to increase HIV testing uptake among MSM and achieve the goal of diagnosing 95% of individuals infected with HIV by 2030.

## 1 Introduction

HIV infection remains a major global public health challenge. According to the latest report from the Joint United Nations Programme on HIV/AIDS (UNAIDS), approximately 40.8 million people were living with HIV worldwide in 2024, of whom only 87% were aware of their infection status (1). Among all populations affected by HIV, men who have sex with men (MSM) bear a disproportionately heavy burden, with an infection risk 26 to 28 times higher than that of the general population (2). In China, the proportion of newly diagnosed HIV infections attributed to male-to-male sexual transmission increased from 2.5% in 2006 to 25.6% in 2022 (3), making MSM a core target population for HIV prevention and control.

The persistence of undiagnosed infections is a critical barrier to epidemic control. Previous studies have demonstrated that approximately 37.6% of new HIV infections are transmitted by undiagnosed individuals (4), who are more likely to engage in high-risk sexual behaviors and are often diagnosed at an advanced stage of infection (5). This not only facilitates secondary transmission during the undiagnosed period but also increases mortality, and healthcare costs (6). Both the World Health Organization (WHO) and China’s HIV prevention and treatment guidelines explicitly recommend that MSM undergo HIV testing every 6 months. To expand HIV testing coverage, China has implemented multiple testing strategies, including organization-based testing, self-testing, peer-driven test kit distribution, and internet-based testing services (7). However, despite these efforts, more than 30% of MSM in China have never been tested for HIV, and the coverage of first-time testing remains far below the levels required for effective epidemic control (8, 9).

Research indicates that young MSM with low perceived HIV risk, limited knowledge of pre-exposure prophylaxis (PrEP), and no history of sexually transmitted infections (STI) are more likely to have never been tested for HIV (10, 11). Notably, first-time testers exhibit a significantly higher HIV prevalence than repeat testers, often accompanied by high-risk sexual behaviors and a discrepancy between risk perception and actual behavior (9). Chongqing and Chengdu, two major cities in southwest China, have been heavily affected by the HIV epidemic among MSM, with cross-sectional surveys reporting HIV prevalence rates as high as 19.7% in Chongqing (12) and 10.2% in Chengdu (13) during peak periods. Although HIV testing rates among MSM in these cities have improved in recent years, a considerable proportion of MSM have yet to establish regular testing habits, making the promotion of first-time testing an urgent challenge.

Although numerous studies have examined factors influencing HIV testing among MSM, most have focused on testing coverage or repeat testing behaviors, while research specifically addressing first-time HIV testing remains relatively limited, particularly in high HIV prevalence settings such as Chongqing and Chengdu. Therefore, this study aims to analyze the factors influencing first-time HIV testing among MSM in these two cities, providing an evidence-based foundation for developing targeted testing promotion strategies. Special emphasis will be placed on identifying high-risk individuals who have never been tested, thereby improving HIV testing coverage among MSM and contributing to the goal of diagnosing 95% of people living with HIV by 2030.

## 2 Materials and methods

### 2.1 Study design and setting

This cross-sectional study was conducted in Chongqing and Chengdu from August to December 2024. Participants were recruited through community-based organizations (CBOs) using convenience sampling. The study received approval from the Ethics Committee of the Chongqing Center for Disease Control and Prevention (approval number: KY-2024-006-2).

### 2.2 Participant

The inclusion criteria for participants were as follows: (1) aged 18 years or older; (2) residing in Chongqing or Chengdu; (3) having engaged in oral or anal sex with other men within the past year; and (4) voluntarily participating in the study with signed informed consent. Participants with serious medical conditions that precluded questionnaire completion (e.g., active malignancy, acute infections, severe mental illness, or cognitive impairment) were excluded.

### 2.3 Study size

The sample size was determined using the formula $n=Z^{2}\cdot p\cdot (1-p)/E^{2}$ , where *p* is the estimated proportion of the behavior of interest. Based on previous surveys, the proportion of MSM reporting no prior HIV testing ranged from 12.9 to 30.1% (14, 15). In this study, *p* was set at 30%, with *α* = 0.05 and a permissible error *E* = 0.05. Under these parameters, a minimum of 323 participants was required. A total of 516 MSM were enrolled. After excluding two participants due to missing data on key sexual behaviors, 514 valid responses were retained for analysis.

### 2.4 Measurement and variables

A structured questionnaire was used to collect data, covering sociodemographic characteristics (age, ethnicity, education level, occupation, monthly income), sexual behavior characteristics (sexual orientation, methods of seeking sexual partners, anal and oral sex with male partners, number of sexual partners, engagement in group sex, sex with female partners in the past month, and history of STI in the past year), as well as the use of new psychoactive substances (NPS) and HIV testing history.

First-time HIV tester was defined as individuals who self-reported had never undergone any HIV testing in their lifetime prior to the study. Participants reported a prior testing history were classified as non-first-time testers (15). NPS use was defined as lifetime use of at least one of the following substances: rush poppers, methamphetamine, marijuana, cocaine, heroin, ketamine, amphetamine or ecstasy. History of STI was defined as a physician-diagnosed presence of one or more of the following conditions in the past year: syphilis, gonorrhea, condyloma acuminatum, genital herpes, or genital *Chlamydia trachomatis* infection.

### 2.5 Bias control

Prior to administering the formal questionnaire, staff from community-based organizations received training through online meetings, which covered participant screening and standardized procedures for questionnaire completion. Prior to the survey, investigators explained the study purpose to participants, emphasized that no identifiable information (e.g., name, ID number, residential address) would be collected, and assured strict confidentiality of all data, which would be used solely for scientific research. During the survey, trained investigators conducted face-to-face interviews with participants in a private and quiet setting using a structured questionnaire. Each questionnaire took approximately 10 to 15 min to complete.

### 2.6 Statistical analysis

Continuous variables were described as mean ± standard deviation or median (interquartile range), depending on their distributional characteristics. Categorical variables were presented as frequencies and percentages. Chi-square tests were used to compare differences in the distribution of sociodemographic and behavioral characteristics. Variables with a *p*-value < 0.10 in univariate analysis were entered into a multivariate logistic regression model to identify factors associated with first-time HIV testing. Odds ratios (ORs) and their 95% confidence intervals (CIs) were calculated. Before constructing the multivariate logistic regression model, multicollinearity among the included independent variables was assessed for each predictor using the variance inflation factor (VIF). Data entry was performed using Excel 2016, and statistical analyses were conducted using R version 4.4.1. A two-sided significance level of *α* = 0.05 was adopted.

## 3 Results

### 3.1 Demographic characteristics

A total of 516 MSM were recruited, of whom two were excluded due to extensive missing data on key sexual behaviors. The final analysis included 514 participants. Among them, 156 (30.4%) were first-time HIV testers. The median age of participants was 35.0 years (interquartile range: 28.0–46.0), with approximately half aged over 35 years. The majority were Han Chinese (97.1%), had a college education or higher (63.4%), were employed (46.5%), were unmarried (61.5%), had a monthly income of 3,000–10,000 RMB (65.0%), and self-identified as homosexual (74.5%, Table 1).

**Table 1:** Socio-demographic and behavioral characteristics among men who have sex with men (*N* = 514).

Variables		Total *n* (%)	First-time HIV testing (*n* = 156) *n* (%)	Non-first-time testing (*n* = 358) *n* (%)	*p-*value
Age (years)	<25	71 (13.8)	30 (19.2)	41 (11.5)	0.042
	25–35	184 (35.8)	48 (30.8)	136 (38.0)	
	>35	259 (50.4)	78 (50.0)	181 (50.6)	
Ethnicity	Han	499 (97.1)	152 (97.4)	347 (96.9)	1.000
	Minorities	15 (2.92)	4 (2.56)	11 (3.07)	
Education	Junior high school and below	67 (13.0)	21 (13.5)	46 (12.8)	0.052
	High school	121 (23.5)	47 (30.1)	74 (20.7)	
	College or above	326 (63.4)	88 (56.4)	238 (66.5)	
Occupation	Company employee	239 (46.5)	74 (47.4)	165 (46.1)	0.075
	Student	43 (8.37)	20 (12.8)	23 (6.42)	
	Unemployed	49 (9.53)	14 (8.97)	35 (9.78)	
	Others	183 (35.6)	48 (30.8)	135 (37.7)	
Monthly income (RMB)	<3000	123 (23.9)	34 (21.8)	89 (24.9)	0.754
	3000–10000	334 (65.0)	104 (66.7)	230 (64.2)	
	>10000	57 (11.1)	18 (11.5)	39 (10.9)	
Sexual orientation	Others	131 (25.5)	43 (27.6)	88 (24.6)	0.546
	Homosexual	383 (74.5)	113 (72.4)	270 (75.4)	
Method to seek sexual partner	Online	389 (75.7)	127 (81.4)	262 (73.2)	0.060
	Offline	125 (24.3)	29 (18.6)	96 (26.8)	
Anal sex with male partners^a^	No	212 (41.2)	58 (37.2)	154 (43.0)	0.255
	Yes	302 (58.8)	98 (62.8)	204 (57.0)	
Oral sex with male partners^a^	No	177 (34.4)	54 (34.6)	123 (34.4)	1.000
	Yes	337 (65.6)	102 (65.4)	235 (65.6)	
Number of sexual partners^a^	≤1	394 (76.7)	125 (80.1)	269 (75.1)	0.265
	≥2	120 (23.3)	31 (19.9)	89 (24.9)	
Engaged in group sex^a^	No	482 (93.8)	152 (97.4)	330 (92.2)	0.039
	Yes	32 (6.2)	4 (2.6)	28 (7.8)	
Sex with female partners^a^	No	454 (88.3)	141 (90.4)	313 (87.4)	0.418
	Yes	60 (11.7)	15 (9.62)	45 (12.6)	
New psychoactive substance use	No	453 (88.1)	130 (83.3)	323 (90.2)	0.038
	Yes	61 (11.9)	26 (16.7)	35 (9.78)	
Sexually transmitted infections (STI)^b^	No	498 (96.9)	155 (99.4)	343 (95.8)	0.049
	Yes	16 (3.1)	1 (0.6)	15 (4.2)	

a In the past month.

b In the past year.

### 3.2 Sexual behaviors

Among the participants, 75.7% sought sexual partners online. In the past month, 58.8% engaged in anal sex, 65.6% engaged in oral sex, 23.3% had two or more sexual partners, and 6.2% reported engaging in group sex; 11.7% had heterosexual sex during the past month. Within the past year, 3.1% had been diagnosed with STI, and 11.9% reported a history of NPS use (Table 1).

### 3.3 HIV testing status

There was a statistically significant difference in age groups between first-time and non-first-time testers (*p* < 0.05). Compared to non-first-time testers, first-time testers had a higher proportion of participants who did not engage in group sex in the past month (*p* = 0.039), a higher proportion with no STI in the past year (*p* = 0.049), and a higher rate of previous NPS use (*p* = 0.038, Table 1).

### 3.4 Factors associated with first-time HIV testing among MSM

Multicollinearity among all independent variables was assessed using VIF prior to constructing the multivariate logistic regression model, and all VIF values were less than 5, indicating no significant multicollinearity. The results showed that MSM with a college degree or higher are less likely to be first-time HIV testers than those with junior high school education or below (adjusted odds ratio [aOR] = 0.46, 95% CI: 0.23–0.93). Participants who did not engage in group sex in the past month were 3.33 times more likely to receive their first HIV test than those who reported group sex (aOR = 3.22, 95% CI: 1.08–9.56). Those with an STI infection in the past year had a significantly lower likelihood of first-time testing (aOR = 0.12, 95% CI: 0.02–0.98; Table 2).

**Table 2:** Factors associated with first-time HIV testing among men who have sex with men.

Variables		aOR (95%CI)	*p*-value
Age (years)	>35 (reference)	1.00	
	<25	1.47 (0.65–3.32)	0.354
	25–35	0.91 (0.56–1.49)	0.711
Education	Junior high school and below (reference)	1.00	
	High school	1.17 (0.60–2.30)	0.641
	College or above	0.46 (0.23–0.93)	0.030
Occupation	Company employee (reference)	1.00	
	Student	1.41 (0.56–3.51)	0.466
	Unemployed	0.78 (0.38–1.59)	0.488
	Others	0.70 (0.42–1.15)	0.161
Method to seek sexual partner	Online (reference)	1.00	
	Offline	0.64 (0.38–1.08)	0.095
Engaged in group sex^a^	Yes (reference)	1.00	
	No	3.22 (1.08–9.56)	0.035
New psychoactive substance use	No (reference)	1.00	
	Yes	1.74 (0.96–3.14)	0.068
Sexually transmitted infections (STI)^b^	No (reference)	1.00	
	Yes	0.12 (0.02–0.98)	0.048

a In the past month.

b In the past year.

## 4 Discussion

First-time HIV testing is the crucial initial step for early detection, intervention, treatment, and prevention of secondary HIV transmission. It also serves as a fundamental prerequisite for achieving the global “95-95-95%” targets for HIV prevention and treatment (16). In this study, 30.4% of MSM were first-time HIV testers, a proportion lower than that reported in Nepal (52.5%) (17) and Brazil (50.5%) (18), broadly consistent with Malaysia (33.8%) (10), but higher than in the United States (15.0%) (19). Compared with domestic studies, our findings are similar to those reported in Tianjin (30.1%) (14) and Zhuhai (29.4%) (11), yet considerably higher than those reported in Foshan, Guangdong Province (22.6%) (20), Beijing (19.5%) (21) and Zhejiang Province (12.9%) (15). These regional variations within China can be attributed to several factors. First, variations in sampling methods and recruitment venues may play a role. Venue-based recruitment (e.g., in bathhouses or community-based organizations) often reaches MSM with lower prior testing coverage, whereas internet-based surveys may reach a higher proportion of individuals with prior testing experience (22). Second, these differences may be associated with regional disparities in economic development and access to testing services. Economically developed regions typically have better public health infrastructure and denser HIV testing networks, which facilitate higher testing accessibility among MSM, thereby leading to lower proportions of first-time testers. The high first-time HIV testing rate among MSM in the Chengdu-Chongqing region reflects that a substantial proportion of this population has not been effectively covered by existing testing networks. This situation underscores the urgency of expanding testing coverage and improving service accessibility to achieve the goal of diagnosing 95% of people living with HIV by 2030.

Consistent with previous studies (23–25), we found that MSM with a college degree or higher are less likely to be first-time HIV testers than those with junior high school education or below. This may be attributed to the fact that MSM with higher educational attainment possess stronger health literacy, actively seek authoritative information, have a more accurate perception of HIV risk, and are more likely to translate knowledge into testing behavior (24, 26). Moreover, higher-educated MSM are more likely to integrate into MSM communities and NGO networks, thereby gaining greater awareness of HIV testing channels (23). Previous studies have also shown that higher-educated MSM tend to prefer more private testing options, such as private clinics or internet-based self-testing, rather than community testing sites, whereas lower-educated MSM often lack awareness of these alternative channels (27). Given these findings, HIV prevention and control efforts should prioritize lower-educated MSM as a key target population for testing promotion, utilizing peer education outreach, mobile testing services, self-testing promotion, and internet-based approaches to improve testing coverage in this subgroup.

We also observed that MSM who had not engaged in group sex within the past month were more likely to be first-time HIV testers. For MSM, group sex is a high-risk activity that typically increases an individual’s risk perception and prompts them to seek testing to confirm their HIV status (28). However, the findings of this study indicate that MSM who have recently engaged in group sex may exhibit distinct testing behavior patterns. First, MSM who recently engaged in group sex may have already established a habit of regular testing or been tested previously. A study by Wei et al. (29) in Shenzhen, China, found that MSM who engage in group sex are more likely to have undergone HIV testing in the past year. Second, MSM who participate in group sex are more likely to be reached by intervention programs and thus become repeat testers, making them less likely to appear as first-time testers. Third, group sex is often associated with deeper involvement in the gay community, and such community engagement facilitates the dissemination of testing information and promotes testing behavior. Conversely, MSM who have not yet developed testing habits and have limited engagement in high-risk social networks may be less likely to engage in group sex and more likely to be first-time testers. Future prospective cohort studies combined with qualitative interviews are needed to further explore the dynamic relationship between group sex and first-time HIV testing.

MSM who contracted STI in the past year were less likely to be first-time HIV testers (11). This association may reflect the role of STI as a catalyst for HIV testing: STI infection prompts MSM to seek medical care, during which they undergo HIV testing. Furthermore, a history of STI infection may increase an individual’s awareness of sexual health and encourage the establishment of regular testing habits (30). The findings of this study indicate only an association between the two, and the causality remains to be established through longitudinal follow-up studies.

This study has several limitations. First, due to the cross-sectional nature of this study, we cannot establish a causal relationship between first-time HIV test and other significantly associated variables, such as STI history in the last year and group sex in the past month. The current findings presented here reflect statistical associations only. Prospective cohort studies are needed to clarify the causal direction between first-time HIV testing and these independent variables. Second, participants were recruited through convenience sampling via CBOs in this study, which may introduce selection bias. As most participants recruited via CBOs have close ties to health services, the sample insufficiently captures MSM with low health literacy, low willingness to test for HIV, and high levels of concealment. Thus, the proportion of first-time testers may be underestimated, and the generalizability of the findings may be limited. Given the practical challenges of implementing probability sampling among this population, CBO-based convenience sampling remains a common approach in current epidemiological studies of MSM in China. Future research should integrate multiple strategies, such as online respondent-driven sampling and venue-based sampling, to reach hidden populations and reduce selection bias. Third, the data were based on self-reports, which may be subject to social desirability and recall biases, particularly for sensitive questions regarding sexual behaviors and drug use. Although a series of procedures were implemented during field surveys to mitigate reporting bias, such bias cannot be completely eliminated. Fourth, a range of factors may affect first-time HIV testing, including risk perception, HIV/AIDS knowledge, awareness of pre-exposure prophylaxis, HIV-related stigma, and testing accessibility. Given that these factors were not collected in the present study, future research should include them to conduct a more comprehensive analysis of the determinants of initial HIV testing.

## 5 Conclusion

In this study, approximately one-third (30.4%) of MSM had never been tested for HIV. MSM with a college degree or higher, and a history of STI in the past year were negatively associated with first-time testing, whereas the absence of group sex in the past month was associated with an increased likelihood of first-time testing. We recommend enhanced, targeted interventions for lower-educated MSM, along with integrated HIV and STI testing services, to increase HIV testing uptake among MSM and achieve the goal of diagnosing 95% of individuals infected with HIV by 2030.

## Data Availability

The raw data supporting the conclusions of this article will be made available by the authors, without undue reservation.
